# A Novel Recombinant FAdV-4 Virus with Fiber of FAdV-8b Provides Efficient Protection against Both FAdV-4 and FAdV-8b

**DOI:** 10.3390/v14020376

**Published:** 2022-02-11

**Authors:** Hao Lu, Quan Xie, Wei Zhang, Jianjun Zhang, Weikang Wang, Mingjun Lian, Zhehong Zhao, Dan Ren, Songhua Xie, Yun Lin, Tuofan Li, Yaru Mu, Zhimin Wan, Hongxia Shao, Aijian Qin, Jianqiang Ye

**Affiliations:** 1 Key Laboratory of Jiangsu Preventive Veterinary Medicine, Key Laboratory for Avian Preventive Medicine, Ministry of Education, College of Veterinary Medicine, Yangzhou University, Yangzhou 225009, China; luhao79@outlook.com (H.L.); drsheer2017@163.com (Q.X.); wwkgyy@sina.com (W.W.); l17863973710@163.com (M.L.); zhehongzhao@163.com (Z.Z.); muzili971010@163.com (D.R.); XieSHsequence@163.com (S.X.); lyun1994@126.com (Y.L.); 007619@yzu.edu.cn (T.L.); mx120190733@yzu.edu.cn (Y.M.); wanzm@yzu.edu.cn (Z.W.); hxshao@yzu.edu.cn (H.S.); 2 Jiangsu Co-Innovation Center for Prevention and Control of Important Animal Infectious Diseases and Zoonoses, Yangzhou 225009, China; 3 Joint International Research Laboratory of Agriculture and Agri-Product Safety, Yangzhou University, Yangzhou 225009, China; 4 Institutes of Agricultural Science and Technology Development, Yangzhou University, Yangzhou 225009, China; 5 Sinopharm Yangzhou VAC Biological Engineering, Yangzhou 225009, China; zhangwei3527408@126.com (W.Z.); yzjjz@126.com (J.Z.)

**Keywords:** FAdV-4, FAdV-8, CRISPR-Cas9, recombinant virus, pathogenicity, inactivated vaccine, protection

## Abstract

Since 2015, the outbreaks of hydropericardium-hepatitis syndrome (HHS) and inclusion body hepatitis (IBH) caused by the highly pathogenic serotype 4 fowl adenovirus (FAdV-4) and serotype 8 fowl adenovirus (FAdV-8), respectively, have caused huge economic losses to the poultry industry. Although several vaccines have been developed to control HHS or IBH, a recombinant genetic engineering vaccine against both FAdV-4 and FAdV-8 has not been reported. In this study, recombinant FAdV-4 expressing the fiber of FAdV-8b, designated as FA4-F8b, expressing fiber of FAdV-8b was generated by the CRISPR-Cas9 and homologous recombinant techniques. Infection studies in vitro and in vivo revealed that the FA4-F8b replicated efficiently in LMH cells and was also highly pathogenic to 2-week-old SPF chickens. Moreover, the inoculation of inactivated the FA4-F8b in chickens could not only induce highly neutralizing antibodies, but also provide efficient protection against both FAdV-4 and FAdV-8b. All these demonstrate that the inactivated recombinant FA4-F8b generated here can act as a vaccine candidate to control HHS and IBH, and FAdV-4 can be an efficient vaccine vector to deliver foreign antigens.

## 1. Introduction

Fowl adenovirus (FAdV) belongs to the family *Adenoviridea* and genus *Aviadenovirus* [1]. Based on the profile of restriction enzyme digestion and sera cross-neutralization assay, FAdV is clustered into five species (FAdV-A~E) with 12 serotypes (FAdV-1~8a,8b~11) [2,3,4]. Epidemiological studies show that FAdV has spread worldwide and cause huge economic loss to the poultry industry. Chickens infected with FAdV generally show subclinical symptoms, while the acute infection of FAdV mainly causes inclusion body hepatitis (IBH), hepatitis-pericardial effusion syndrome (HHS) and gizzard erosion and ulceration (GEU) [3,5,6]. Notably, clinical data indicate that different species or serotypes of FAdV can induce distinct pathogenic symptoms. HHS is mainly caused by the highly pathogenic FAdV-4 [7,8], whereas FAdV-8a, 8b and 11 are mainly related to IBH [6,9]. In the past, HHS and IBH only occurred sporadically in China. However, since 2015, the outbreaks of HHS and IBH in chicken flocks have widely spread in China [7,10,11,12]. Molecular and epidemiological data reveal that FAdV-4 and FAdV-8 are the dominant serotypes endemic in China [7,13,14,15]. Although several inactivated or subunit vaccines have been developed against FAdV-4 or FAdV-8 [11,12,16,17], an inactivated and recombinant genetic engineering vaccine against both FAdV-4 and FAdV-8 has not been reported.

Fiber plays an extremely important role in triggering virus infection and inducing neutralizing antibodies and can be used as an efficient protective immunogen for developing a vaccine against FAdVs [12,18,19]. In our previous study, an attenuated FAdV-4 recombinant virus FAd4-EGFP with the expression of EGFP-Fiber-2 fusion protein was generated via the CRISPR-Cas9 technique [20]. In this study, a similar strategy was used to generate an FAdV-4 recombinant virus FA4-F8b carrying the fiber of FAdV-8b. An in vivo study showed that, although FA4-F8b was lethal to SPF chicken, the inactivated FA4-F8b could induce highly neutralizing antibodies against both FAdV-4 and FAdV-8b and provide efficient protection against both FAdV-4 and FAdV-8b.

## 2. Materials and Methods

### 2.1. Cells, Viruses, Antibodies and Plasmids

The FAdV-4 strain SD and the FAdV-8b strain JSSQ15 were isolated and stored in our laboratory and propagated in leghorn male hepatoma (LMH) cells [7]. The FAdV-8a strain AH720 was kindly provided by Professor Hongjun Chen. The recombinant virus FA4-EGFP was generated by our laboratory [20]. LMH cells from the American strain collection center (ATCC) were cultured in Dulbecco Modified Eagle Medium/F12 (Gibco, NY, USA) containing 10% fetal bovine serum, and placed in a 5% CO_2_ incubator at 37 °C. Monoclonal antibody (mAb) 5F10 against fiber of FAdV-8b, mAb 3B5 against the Fiber-1 of FAdV-4, and chicken sera against FAdV-4 were generated and stored in our laboratory. mAb 1B5 against the hexon of FAdVs, mAb 1C9 against Fiber-2 of FAdV-4, and chicken sera against FAdV-8a strain AH720 were kindly provided by Professor Hongjun Chen. The pMD19- HAL-EGFP-F2-HAR simple vector was constructed and stored in our laboratory [20].

### 2.2. Construction of sgRNA and Donor Plasmids

These sgRNA-L and sgRNA-R (Table 1) targeting EGFP in the FA4-EGFP genome were designed by Zhang Lab through the website [21,22] and inserted into the lentiCRISPR v2 vector. Donor plasmid containing the *fiber* sequence of FAdV-8b located at the C-terminus of Fiber-1 and the *N*-terminus of Fiber-2 of FAdV-4 was constructed by homologous recombination based on the pMD19-HAL-EGFP-F2-HAR vector, which was named the pMD19-HAL-F-F2-HAR vector [20]. The primers used for constructing donor plasmid are listed in Table 2.

### 2.3. Generation of the Recombinant FA4-F8b

LMH cells were first transfected with the sgRNA targeting both ends of the EGFP-Fiber-2 gene with 2 μg of each sgRNA and 4 μg of the donor plasmid, and the LMH cells were infected with FA4-EGFP at a multiplicity of infection (MOI) of 0.1 at 24 h post-transfection (hpt). The target infected LMH cells could be observed showing cytopathic effect without green fluorescence at 24 h post-infection (hpi) using a fluorescence microscope. The recombinant virus, named FA4-F8b, was purified by limiting the dilution and virus plaque assay.

### 2.4. Identification the Stability and Growth Properties of the FA4-F8b in LMH Cells

The insertion of the *fiber* gene was detected by PCR using primers located at the recombinant arms: Ad8T-F (5′-TATCAGGGTTACGTCTACTCCC-3′) and Ad8T-R (5′-GCTTCGGTCTCGGGCTTCCCGT-3′). To assess the replication capacity of the FA4-F8b, the growth kinetics of FAdV-4 and the FA4-F8b were detected in LMH cells at an MOI of 0.01, and the viruses were harvested at 24, 48, 72, 96 and 120 hpi, respectively. Then, the TCID_50_ of the harvested viruses were determined by IFA and calculated by the Reed-Muench method.

### 2.5. Western Blot Assay

The infected LMH cells were collected and lysed in lysis buffer (CoWin Biosciences, Taizhou, China) with protease and phosphatase inhibitor cocktail (New Cell and Molecular Biotech, Suzhou, China). The lysed cells were added with loading buffer and boiled for 10 min. After transient dissociation, the supernatant of lysed cells was subjected to SDS-PAGE and transferred to nitrocellulose (NC) membrane at constant pressure. The NC membrane was blocked with PBST containing 5% skimmed milk at room temperature (RT) for 2 h. The membrane was incubated with the primary antibody diluted in PBST containing 5% skimmed milk at 4 °C overnight. After washing with PBST for three times, the NC membrane was reacted with the secondary antibody labeled with HRP at RT for 1 h. After three washes with PBST, the membrane was treated with ECL substrate (CoWin Biosciences, Taizhou, China) and developed with an automatic imaging system (Tanon 5200).

### 2.6. IFA

The infected LMH cells were fixed with prechilled acetone: ethanol (3:2 *v*/*v*) mixture for 5 min at RT and washed with PBS once. The cells were then incubated with the primary antibody for 45 min at 37 °C. After being washed three times with PBS, the cells were incubated with the diluted second antibody conjugated with FITC for another 45 min at 37 °C. After being washed another three times with PBS, the cells were observed under invert fluorescence microscopy.

### 2.7. Pathogenicity of the Recombinant FA4-F8b in SPF Chickens

To evaluate the pathogenicity of FA4-F8b in SPF chicken, a total of 30 4-week-old SPF chickens were randomly divided into three groups (10 chickens in each group). Chickens were intramuscularly infected with 10^6^ TCID_50_ of FAdV-4 (positive control group) or FA4-F8b (experiment group). Chickens inoculated with 1% culture medium were set as the negative control group. The clinical symptoms and mortality of the infected chickens were monitored daily, and the necropsy examination for deceased chickens was carried out.

### 2.8. Preparation of Inactivated FA4-F8b Vaccine

To inactivate FA4-F8b virus, the recombinant virus was inactivated by adding 0.3% formaldehyde at 37 °C for 24 h. The inactivated FA4-F8b was mixed at a 1:3 ratio with oil adjuvant to make an inactivated vaccine and the culture medium was treated with the same way as a control. The final viral dose of the inactivated oil-emulsion FA4-F8b vaccine was 10^6^ TCID_50_ in 0.4 mL per chicken.

### 2.9. Immunization and Challenge

A total of 175 7-day-old chickens were randomly divided into 7 groups as summarized in Table 3, and the chickens in Group 2, 4, and 6 were vaccinated intramuscularly with 0.4ml of the inactivated FA4-F8b/chicken. The chickens in Groups 1, 3, 5, and 7 (challenge controls) were inoculated with a culture medium/adjuvant mixture. At 28 days post vaccination (dpv), the chickens in Group 2 (vaccine/challenge FAdV-8b) and Group 3 (challenge control FAdV-8b) were challenged with 10^6^ TCID_50_ of FAdV-8b, the chickens in Group 4 (vaccine/challenge FAdV-8a) and Group 5 (challenge control FAdV-8a) were challenged with FAdV-8a, the chickens in Group 6 (vaccine/challenge FAdV-4) and Group 7 (challenge control FAdV-4) were challenged with FAdV-4, and the chickens in Group 1 (adjuvant only) were injected with PBS. At 1, 3, 5, 7, 9, and 11 days post-challenge (dpc), the cloacal swabs were collected. Three chickens from each group were euthanized at 3, 5, 7, and 9 dpc and the liver, the spleen and the kidney were collected for viral titration. The clinical symptoms and mortality of the infected or challenged chickens were monitored daily. Additionally, the sera were collected from all the chickens at 7, 14, 21, and 28 dpv for detection of the neutralizing antibodies. These animal experiments were carried out in accordance with the experimental animal guidelines and protocol (SYXY-20) approved by the Animal Care and Use Committee of Yangzhou University (Yangzhou, China). At the end of all animal experiments, the experimental chickens were euthanized with carbon dioxide.

### 2.10. Virus Neutralization Test (VNT)

Sera of the vaccinated chickens (Groups 1–7) collected at 7, 14, 21, and 28 dpv were tested for neutralizing the antibody against the three viruses (FAdV-8b, FAdV-8a, and FAdV-4) as previously described [23].

### 2.11. Titration of Viral Titer in Organs and Cloacal Swabs

The liver, spleen and kidney collected were homogenized and treated with penicillin-streptomycin at 37 °C for 1 h and centrifuged at 4 °C to obtain the supernatant. The collected cloacal swabs were placed in 800 μL of PBS. After three freeze–thaw cycles, the swabs were treated with the above method. The virus-containing supernatants were inoculated into LMH cells by serial dilution. The infected LMH cells were fixed and tested by IFA using mAb 5F10 against fiber of FAdV-8b, chicken sera against FAdV-8a and mAb 3B5 against Fiber-1 of FAdV-4, and the TCID_50_ of these supernatants was determined by the Reed–Muench method.

### 2.12. Statistical Analysis

All data were shown as the means ± SD. Additionally, statistical analysis in this study was executed with a Student’s test or one-way ANOVA *t*-test using GraphPad 6 software. *p* < 0.05 was considered statistically significant. *, **, *** and **** demonstrate *p* < 0.05, 0.01, 0.001, and 0.0001, respectively.

## 3. Results

### 3.1. Generation of a Recombinant Virus FA4-F8b Expressing the Fiber of FAdV-8b

**Figure 1:**
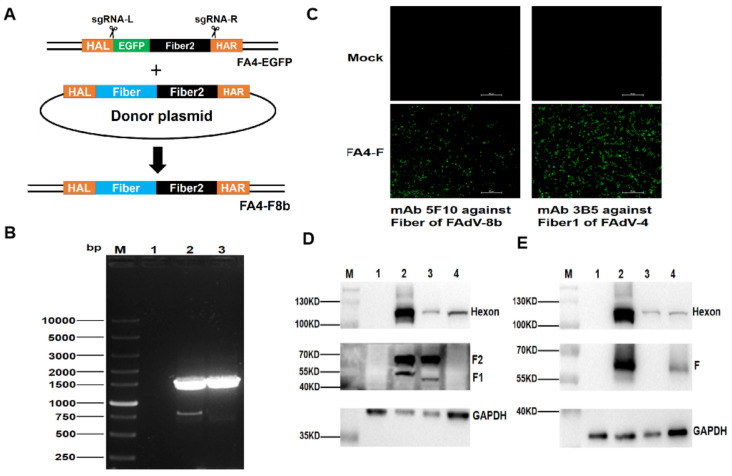
Generation of the recombinant FA4-F8b expressing fiber of FAdV-8b. (**A**)The homology-dependent knock-in strategy for generating the recombinant virus FA4-F8b using the CRISPR-Cas 9 system. LMH cells were simultaneously transfected with two sgRNAs and donor plasmid. Then, the LMH cells were infected with FA4-EGFP at 24 hpt. The recombinant virus FA4-F8b was then purified by viral plaque assay and limiting dilution assay. (**B**) The purified FA4-F8b was detected by PCR. The LMH cells (Lane 1), the unpurified FA4-F8b (Lane 2) and the purified FA4-F8b (Lane 3) were detected using specific primers (Ad8T-F and Ad8T-R). (**C**) The purity and the expression of fiber of the purified FA4-F was detected by IFA. LMH cells were infected with the purified FA4-F8b. mAb 3B5 against Fiber-1 of FAdV-4 and mAb 5F10 against fiber of FAdV-8b were used to test the purified FA4-F8b, respectively. The uninfected LMH cells were set as negative control. (**D**) Western blot analysis for the recombinant virus FA4-F8b using PcAb against Fiber-1, mAb against Fiber-2 and mAb against the Hexon of FAdVs. LMH cells (NC), LMH cells infected with the purified recombinant virus FA4-F8b (Lane 2), the FAdV-4 (Lane 3) and the FAdV-8 (Lane 4) were harvested and lysed, and the lysates were then tested by Western blot. (**E**) Western blot analysis for the recombinant virus FA4-F8b using mAb against fiber of FAdV-8b and mAb against the Hexon of FAdVs. LMH cells (NC), LMH cells infected with the purified recombinant virus FA4-F8b (Lane 2), the FAdV-4 (Lane 3) and the FAdV-8 (Lane 4) were harvested and lysed, and the lysates were then tested by Western blot.

To generate a recombinant virus FAdV-4 carrying the fiber of FAdV-8b, two sgRNAs targeting C- and *N*-terminus of EGFP and *fiber-2* of the FA4-EGFP, respectively, were first designed and cloned into lentiCRISPR v2, and the donor plasmid containing the *fiber* of FAdV-8b and *fiber-2* of FAdV-4 was constructed. The constructed sgRNA and the donor plasmid were then transfected into LMH cells followed by the infection of FA4-EGFP at 24 hpt (Figure 1A). The rescued recombinant virus expressing the fiber of FAdV-8b were purified by serial limit dilution and plague purification and designated as FA4-F8b. To verify whether the sequence of the rescued FA4-F8b was correct, the insertion site of *fiber* of FAdV-8b between *fiber-1* and *fiber-2* of FAdV-4 was confirmed in the rescued FA4-F8b by PCR (Figure 1B). As described in Figure 1C–E, the rescued FA4-F8b could efficiently express the fiber of FAdV-8b, the Fiber-1 and Fiber-2 of FAdV-4 in the infected LMH cells. All these demonstrate that a novel FAdV-4 recombinant virus FA4-F8b efficiently expressing the fiber protein of FAdV-8b is generated. The efficient expression of the three fiber proteins in FA4-F8b is critical for the potential application of FA4-F8b as a vaccine candidate for both FAdV-4 and FAdV-8b.

### 3.2. FA4-F8b Replicated Efficiently In Vitro and Was Highly Pathogenic In Vivo

**Figure 2:**
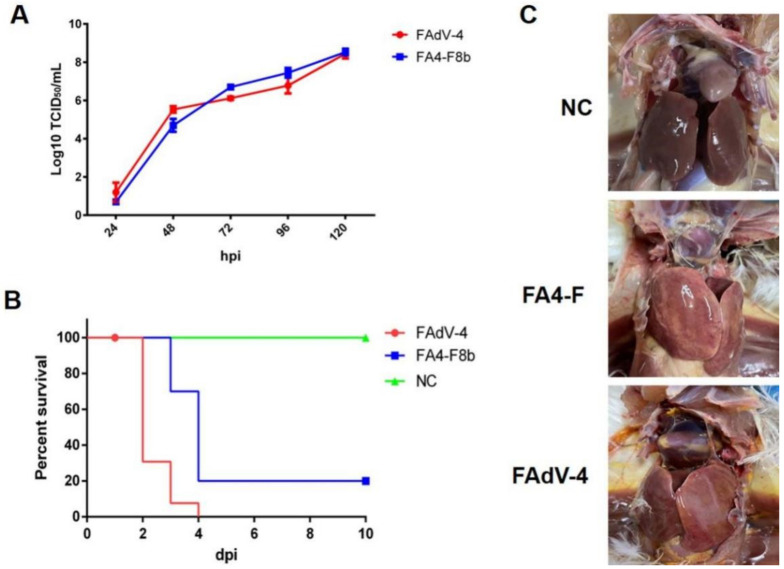
FA4-F8b replicated efficiently in vitro and was pathogenic in vivo. (**A**) LMH cells were infected with FA4-F8b and FAdV-4 at the same dose, respectively, and the viral supernatant collected from the infected LMH cells at the indicated time points were then titrated by TCID_50_. (**B**) SPF chickens were randomly divided into three groups and then inoculated with FA4-F8b, WT FAdV-4, and 1% culture medium, respectively. Percent of survival for these infected chickens was calculated according to the result of the infection study. (**C**) The representative gross lesion in the heart and liver from the chickens infected with FA4-F8b, the chickens with FAdV-4 and the chickens inoculated with 1% culture medium, respectively.

To evaluate the viral replication ability of FA4-F8b, the growth kinetics of the FA4-F8b was compared with the wild-type (WT) FAdV-4 in LMH cells. As described in Figure 2A, both FA4-F8b and FAdV-4 efficiently replicated in LMH cells with very similar replication kinetics, and the peak titer of both viruses could reach to 10^8^ TCID_50_/_mL_ at 5 dpi. In vivo study further demonstrated that FA4-F8b was lethal for 28-day-old SPF chickens. As shown in Figure 2B, the mortality of chickens infected with FAdV-4 was 100% (10/10), whereas the chickens infected with FA4-F8b also reached 80% (8/10). Therefore, in comparison with FAdV-4, the pathogenicity of FA4-F8b was slightly attenuated based on the survival data of the infected chickens. Additionally, in the necropsy examination, all the dead chickens infected with either FAdV-4 or FA4-F8b showed severe hydropericardium syndrome and hepatitis, whereas all of the SPF chickens in the negative control group did not show any clinical signs and pathological symptoms as described in Figure 2C.

### 3.3. Inactivated FA4-F8b Induced Efficiently Neutralizing Antibodies

**Figure 3:**
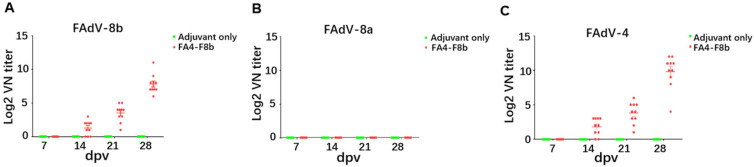
Inactivated FA4-F8b induced efficient neutralizing antibodies against both FAdV-4 and FAdV-8b. The neutralizing activity (NT) of sera against FAdV-8b (**A**), FAdV-8a (**B**), and FAdV-4 (**C**) from the inoculated chickens were tested at 7, 14, 21 and 28 dpv, and then these chickens at 28 dpv were infected with FAdV-8b, FAdV-8a and FAdV-4 at 28 dpv, respectively.

Since FA4-F8b replicated efficiently and was pathogenic in vitro and in vivo, FA4-F8b was inactivated and evaluated for protective efficacy against FAdV-4, FAdV-8b, and FAdV-8a in SPF chickens as described in the materials and methods part. To test whether the inactivated FA4-F8b could induce neutralizing antibody against both FAdV-4, FAdV-8b, and FAdV-8a, the neutralization titer (NT) of sera from chickens immunized with the inactivated FA4-F8b at 7, 14, 21, and 28 dpv were tested. As described in Figure 3, the average NT of sera from these chickens was 0, 1.3, 3.5, and 7.8 against FAdV-8b, 0, 1.8, 3.8 and 9.8 against FAdV-4, and 0, 0, 0, and 0 against FAdV-8a at 7, 14, 21 and 28 dpv, respectively, whereas that in chickens from the negative control could not be detected. All these data demonstrate that the inactivated FA4-F8b can efficiently induce neutralizing antibodies with high titers against both FAdV-4 and FAdV-8b.

### 3.4. Inactivated FA4-F8b Provides Efficient Protection against Both FAdV-8b and FAdV-4

**Figure 4:**
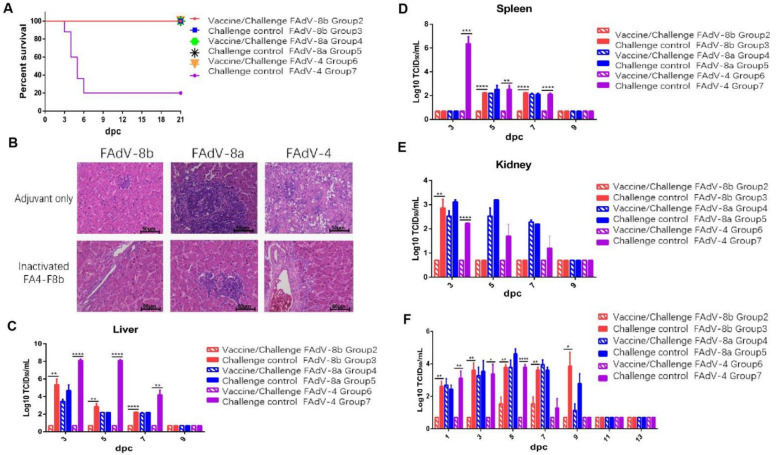
Inactivated FA4-F8b provided efficient protection against both FAdV-4 and FAdV-8b. SPF chickens were randomly divided into 7 groups, then inoculated with inactivated vaccine and adjuvant, respectively. After 28dpv, the chickens were infected with FAdV-8b, FAdV-8a, and FAdV-4, respectively. The clinical symptoms and mortality of the infected chickens were monitored daily, and the liver, spleen, kidney, and cloacal swabs were collected for viral titration at the indicated time points. (**A**) Percent of survival for the challenged chickens. (**B**) Representative histological changes in liver tissues from the challenged control chickens and the challenged chickens previously inoculated with FA4-F8b. Viral loads in the liver (**C**), spleen (**D**), and kidney (**E**) tissues from the challenged chickens. Viral shedding in cloacal swabs (**F**) from the challenged chickens (*, **, *** and **** indicate *p* < 0.05, 0.01, 0.001, and 0.0001, respectively).

To evaluate whether the inactivated FA4-F8b could provide protection against FAdV-8 and FAdV-4, the chickens immunized with the inactivated FA4-F8b were challenged with the FAdV-8 and FAdV-4, respectively. The clinical signs and mortality of the challenged chickens were monitored daily. After challenge, the chickens in Group 7 showed signs of depression, loss of appetite and huddling together with ruffled feathers, and the chickens in other groups did not show significant clinical signs. For the survival data, as shown in Figure 4A, the chickens in Group 7 (control chickens challenged with FAdV-4) began to die at 3 dpc, and finally reached 80% mortality within 6 dpc, whereas the chickens in the other groups all survived. Moreover, the typical lesion of HHS could be observed after necropsy for the dead chickens in the control Group 7, whereas the chickens previously inoculated with FA4-F8b did not show any signs of HHS (data not shown). The histopathological analysis further demonstrated that the degeneration and necrosis of hepatocytes and the intranuclear inclusion bodies in hepatocytes were observed in the chickens from Group 7. In addition, the lymphatic vesicles and inflammatory cell infiltration were observed in the hepatic cells in the chickens from Group 3 (challenge–control FAdV-8b), Group 4 (vaccine/challenge FAdV-8a) and Group 5 (Challenge–control FAdV-8a), whereas no obvious histopathological symptoms were found in the chickens from the rest groups as shown in Figure 4B. For the determination of viral titer, the liver, spleen, kidney and cloacal swabs of the challenged chickens were collected at indicated time points post challenging. As described in Figure 4C–E, high viral titers in the liver, spleen, and kidney of chickens in Groups 3, 4, 5 and 7 were detected at 3, 5 and 7 dpc, whereas no virus could be detected from these tissues in Group 2 and 6. Similar data were found in the cloacal swab samples from these chickens. As shown in Figure 4F, high viral titers in the cloacal swab of chickens in Groups 3, 4, 5 and 7 were detected at 1, 3, 5, 7 and 9 dpc, whereas no or few viruses could be detected from the cloacal swabs in Groups 2 and 6. All these data demonstrate that the inactivated FA4-F8b can provide efficient protection against both FAdV-4 and FAdV-8b, but not FAdV-8a.

## 4. Discussion

Recently, HPS and IBH caused by FAdV-4 and FAdV-8 have spread globally and resulted in huge economic losses to the poultry industry [8,11,24]. Although several inactivated or recombinant subunit or attenuated vaccines against FAdV-4 or FAdV-8 have been developed [12,17,18,25,26,27,28], a bivalent vaccine against both FAdV-4 and FAdV-8 is still not available for HPS and IBH. Since fiber protein is one of the importantly protective immunogens of FAdV [12,18,19], to generate a bivalent vaccine against both FAdV-4 and FAdV-8, the *fiber* gene of FAdV-8b was inserted into the genome of FAdV-4 to generate a novel FAdV-4 recombinant virus FA4-F8b through CRISPR-Cas9 and homologous recombination techniques as previously described [20]. The recombinant virus FA4-F8b expressing the fiber protein of FAdV-8b showed the similar viral growth kinetics with the wild-type FAdV-4 and also was lethal to SPF chickens. However, the inactivated FA4-F8b could efficiently induce potent neutralizing antibodies against both FAdV-4 and FAdV-8b and protected the challenges of both FAdV-4 and FAdV-8b.

Notably, the recombinant FA4-F8b could efficiently express the fiber of FAdV-8b, and the Fiber-1 and Fiber-2 of FAdV-4 as shown in Figure 1D,E. Since the *fiber* of FAdV-8b was not fused with either *fiber-1* or *fiber-2* of FAdV-4, the molecular weight of *fiber* of FAdV-8b and *fiber-2* of FAdV-4 expressed in FA4-F8b was the same as wild-type FAdV-8b and FAdV-4, respectively. However, the molecular weight of *fiber-1* of FAdV-4 expressed in FA4-F8b was slightly greater than that of *fiber-1* of wild-type FAdV-4 due to the ORF of the *fiber-1* of FAdV-4 in FA4-F8b carrying an addition sequence of 143bp derived from the *N*-terminus of *fiber* of FAdV-8b. The mechanism of the efficient expression of the three fibers in FA4-F8b without an additional promoter needs to be elucidated. Different from the highly attenuated and recombinant virus, FA4-EGFP expressing a fusion protein of EGFP with Fiber-2 [20], FA4-F8b was lethal for SPF chickens. The intact *fiber-2* of FAdV-4 in FA4-F8b might contribute to the lethal phenotype of FA4-F8b, and the fusion of EGFP and *fiber-2* might affect the function of *fiber-2* and result in attenuation. In addition, we found that the purified FA4-F8b passaged in LMH cells for more than 10 passages was very stable with fiber of FAdV-8b and without the reversion into the wild-type FAdV-4 (data not shown).

Different from the highly pathogenic FAdV-4 endemic in China, serotypes FAdV-8a and FAdV-8b were not lethal for SPF chickens. Therefore, the neutralizing antibodies induced and the viral replication and shedding for FAdV-8a and FAdV-8b were tested to evaluate the protective efficacy of FA4-F8b. It is noteworthy that chickens immunized with inactivated FA4-F8b could induce efficient neutralizing antibody and provide protection against both FAdV-4 and FAdV-8b, but not FAdV-8a. As described in Figure 3 and Figure 4, FA4-F8b could not induce detectable neutralizing antibody against FAdV-8a and could not efficiently inhibit the viral replication and shedding of FAdV-8a in tissues and cloaca, respectively. Therefore, although the homology of fiber protein between FAdV-8a and FAdV-8b was about 80%, the protective antigens in the inactivated FA4-F8b against FAdV-4 and FAdV-8b did not show cross-protection against FAdV-8a.

## 5. Conclusions

This is the first demonstration of the generation of a novel lethal FAdV-4 recombinant virus FA4-F8b expressing the fiber of FAdV-8b through the CRISPR-Cas9 technique. The inactivated FA4-F8b can offer efficient protective efficacy against both FAdV-4 and FAdV-8b, highlighting that the inactivated FA4-F8b can be a vaccine candidate for preventing HPS and IBH caused by FAdV-4 and FAdV-8b. The generation of FAdV-4 recombinant virus FA4-F8b expressing the fiber of FAdV-8b with a high virus titer also indicates that FAdV-4 has great potential to be as an efficient vaccine vector for expressing or delivering foreign genes for protecting other pathogens in future.

## Figures and Tables

**Table 1:** List of primers used for sgRNA cloning.

	Sequences of Primers (5′–3′)
sgRNA-L	F: CACCGGGTTACGTCTACTCCCCCAA
	R: AAACTTGGGGGAGTAGACGTAACCC
sgRNA-R	F: CACCGTCTTTATTTGACACGCGGTG
	R: AAACCACCGCGTGTCAAATAAAGAC

**Table 2:** PCR primers for constructing donor plasmid.

PCR Products	Sequences of Primers (5′–3′)
Linear pMD19- HAL-EGFP-F2-HAR	F: CACCGGGTTACGTCTACTCCCCCAA
	R: AAACTTGGGGGAGTAGACGTAACCC
Fiber gene of FAdV-8b	F: CACCGTCTTTATTTGACACGCGGTG
	R: AAACCACCGCGTGTCAAATAAAGAC

**Table 3:** Design of the animal experiment.

Group	Designation	Vaccination	Challenge
1	Negative control	Adjuvant only	- *
2	Vaccine/challenge FAdV-8b	Inactivated FA4-F8b	FAdV-8b
3	Challenge control FAdV-8b	Adjuvant only	
4	Vaccine/challenge FAdV-8a	Inactivated FA4-F8b	FAdV-8a
5	Challenge control FAdV-8a	Adjuvant only	
6	Vaccine/challenge FAdV-4	Inactivated FA4-F8b	FAdV-4
7	Challenge control FAdV-4	Adjuvant only	

* Not applicable.

## Data Availability

All data are included in the manuscript.
